# An investigation of the diet, exercise, sleep, BMI, and health outcomes of autistic adults

**DOI:** 10.1186/s13229-021-00441-x

**Published:** 2021-05-08

**Authors:** Elizabeth Weir, Carrie Allison, Ken K. Ong, Simon Baron-Cohen

**Affiliations:** 1 Autism Research Centre, Department of Psychiatry, University of Cambridge, Douglas House, 18B Trumpington Road, Cambridge, England CB2 8AH; 2 MRC Epidemiology Unit and Department of Pediatrics, Institute of Metabolic Science, University of Cambridge, Cambridge Biomedical Campus,, Cambridge, England CB2 0QQ

**Keywords:** Healthcare, Physical health, Comorbidities, Adult outcomes, Nutrition, Exercise, Sleep

## Abstract

**Background:**

Studies of autistic children suggest that restricted eating, reduced physical activity, and sleep disorders are common; however, no studies attempt to broadly describe the diet, exercise, and sleep patterns of autistic adults or consider relationships between lifestyle behaviors and the widely reported increased risks of obesity and chronic conditions. To address this, the authors developed the largest study of lifestyle patterns of autistic adults and assessed their relationships to body mass index, health outcomes, and family history.

**Methods:**

We administered an anonymized, online survey to *n* = 2386 adults (*n* = 1183 autistic) aged 16–90 years of age. We employed Fisher’s exact tests and binomial logistic regression to describe diet, exercise, and sleep patterns; mediation of seizure disorders on sleep; body mass index (BMI); relationships of lifestyle factors to BMI, cardiovascular conditions, and diabetic conditions; and sex differences among autistic adults.

**Results:**

Autistic adults, and particularly autistic females, exhibit unhealthy diet, exercise, and sleep patterns; they are also more likely to be underweight or obese. Limited sleep duration and high rates of sleep disturbances cannot be accounted for by epilepsy or seizure disorders. Lifestyle factors are positively related to higher risk of cardiovascular conditions among autistic males, even more than family history.

**Limitations:**

Our sample may not be representative of all autistic and non-autistic people, as it primarily comprised individuals who are white, female, have a high school education or higher, and reside in the UK. Our sampling methods may also exclude some individuals on the autism spectrum, and particularly those with moderate to severe intellectual disability. This is a cross-sectional sample that can test for relationships between factors (e.g., lifestyle factors and health outcomes) but cannot assess the direction of these relationships.

**Conclusions:**

Autistic adults are less likely to meet minimal health recommendations for diet, exercise, and sleep—and these unhealthy behaviors may relate to excess risk of cardiovascular conditions. Although the present study can only provide preliminary, correlational evidence, our findings suggest that diet, exercise, and sleep should be considered and further investigated as key targets for reducing the now widely reported and dramatically increased risks of health comorbidity and premature death among autistic individuals compared to others. Physicians should work cooperatively with patients to provide health education and develop individualized strategies for how to better manage challenges with diet, exercise, and sleep.

**Supplementary Information:**

The online version contains supplementary material available at 10.1186/s13229-021-00441-x.

## Background

Autism spectrum conditions (henceforth autism) are lifelong, neurodevelopmental conditions characterized by social and communication difficulties, repetitive behaviors, restricted interests, and differences in cognitive profile, including atypical sensory perception, motor abilities, and intellectual ability [1]. The prevalence of autism is one to two percent in the general population, and this figure has risen over time, likely due to changes in diagnostic criteria and better detection of the condition [2]. There is a sex-bias in autism, with males being diagnosed approximately three to four times more often than females [2]. As autism is lifelong, and prevalence of the condition has increased in recent years, greater numbers of autistic individuals are reaching adulthood; and in turn, this requires greater recognition of the challenges faced by autistic individuals across the lifespan.

Autistic individuals may be more likely to develop a variety of physical and mental health conditions, including Type II diabetes, certain cancers, respiratory conditions, and cardiovascular conditions [3–7], with relatively greater risk for autistic females compared to autistic males [3, 4, 7]; and these conditions may contribute to increased risk of premature mortality seen among autistic individuals [8, 9]. Estimates of risk of premature mortality are alarming, suggesting that autistic individuals die 16–38.5 years younger on a average than expected [8, 9], with greatest risk among autistic females and those with intellectual disability (ID) [9].

It is currently unclear why autistic individuals have a greater health burden; however, lifestyle factors are of particular interest, as autistic individuals are more likely to be overweight or obese [3, 6, 10–15]. Being overweight or obese contributes significantly to risk of developing chronic physical health conditions, including diabetic and cardiovascular conditions [6, 16]. Poor nutrition, limited physical activity, and sleep disorders all appear to be risk factors for obesity among autistic children [17–21]. The authors are not aware of any studies that comprehensively describe the lifestyle factors of autistic adults, despite their potential to be key targets for intervention that improve health outcomes.

Regarding diet, autistic children are more likely to have disordered or atypical eating patterns (including food refusal, limited diet/picky eating, pica, anorexia, avoidant, and restricted food intake disorder (ARFID), binge-eating, etc.), with some estimates suggesting that 70% of autistic children have atypical eating behaviors [22–25]. Individuals with eating disorders are also more likely to be autistic and have higher autistic traits than others [26–29]. Atypical eating behaviors may be self-imposed due to differences in cognitive style (including behavioral inflexibility), increased likelihood for engaging in emotional eating behaviors (particularly among autistic females), and/or sensory sensitivities [22, 30–32]. Dietary choices may also be restricted due to high rates of food allergies and gastrointestinal conditions among autistic individuals [3, 4, 33–36].

Finally, caregivers may impose dietary restrictions as interventions to reduce autism symptoms or challenging behaviors, though there is mixed evidence of their effectiveness [35, 37–39]. Feeding problems and restricted eating of autistic children affect nutrition, which may have knock on effects on growth, autism symptoms, risk of malnutrition or obesity, and health status [22, 40–43]. A few, small-scale studies suggest that these trends may persist into adulthood [44–46]. As yet, there are no large-scale studies that monitor dietary restrictions, nutrition, or eating behavior in autistic adults over the age of 35, or in autistic adults without ID.

It is also clear that autistic children are less likely to engage in physical activity than non-autistic children [14, 20, 47, 48]. Autistic individuals appear to be less interested in exercise due to differences in social motivation, increased screen time, and differences in motor ability [12, 18, 48–54]. Some interventions aimed to increase physical activity may reduce autism symptoms and improve some fitness and metabolic indicators [55–58]. Despite some evidence that autistic children, and particularly autistic girls, engage less in physical activity as they age [12, 59–61], there are currently no large-scale studies that indicate whether patterns of inactivity persist into adulthood, the degree to which autistic adults engage in physical activity, or whether any interventions might be useful in increasing physical activity levels among autistic adults [62].

Regarding sleep, autistic individuals of all ages are more likely to have a variety of sleep disorders and limited sleep duration [4, 5, 47, 63, 64]. Among autistic children, sleep disturbances are associated with symptom severity, younger age, Hispanic ethnicity, higher intellectual quotient (IQ), ADHD, behavioral dysregulation/problems, lower adaptive function, epilepsy/seizures, maternal autistic traits, poorer child mental health, poorer maternal mental health (including anxiety and depression), poorer maternal and paternal physical health, lower caregiver/parental education, and lower family income [51, 64–68]. In a large sample of autistic children, obesity was associated with sleep difficulties and melatonin use, further emphasizing the relationships between sleep quality, metabolism, and markers of overall health within this population [69]. However, there are only three studies and one meta-analysis, all with relatively small sample sizes, that consider specific sleep disorders and/or sleep traits in depth among autistic adults–with some evidence that social impairment and biological sex may be related to sleep dysfunction [63, 64, 70, 71].

Overall, evidence suggests that autistic children and adolescents are unable to meet the minimum recommendations for diet, exercise, and sleep that promote good health and prevent chronic disease; yet, there are no studies of whether or not these patterns are also broadly seen in autistic adults. The current study examines whether obesity-related dietary, exercise, and sleep patterns are seen among autistic adults, as well as whether these lifestyle factors contribute to the elevated risks of chronic diseases seen among autistic adults.

## Methods

We developed an anonymous, self-report survey using a cross-sectional, convenience-sampling framework via Qualtrics. The survey included questions on demographics, a short version of the Autism Spectrum Quotient (a measure of autistic traits, AQ-10) [72], and daily habits (including exercise, diet, sleep, disability, and social/sexual history), as well as personal and family medical histories of common medical conditions. The final sample comprised *n* = 2386 individuals, including *n* = 1183 autistic individuals and *n* = 1203 controls. The mean age of the autistic group was 41.04 years of age (standard deviation = 14.41), and the mean age of the control group was 41.86 years (standard deviation = 15.59). Both the autistic and control samples were predominantly composed of females, white individuals, UK residents, and those without ID; however, there were significant group differences in distributions of sex, ethnicity, education, country of residence, BMI, alcohol use, smoking, and intellectual disability status. Table 1 includes a summary of demographic information for the autistic and control groups.

**Table 1:** Participant demographics

Characteristics	Autism (*n* = 1183)	Controls (*n* = 1203)	p-values (signif. level)
Age (years), mean (SD)	41.04 (14.41)	41.86 (15.59)	0.344
Age (years), categories, *N* (%)			
16–29	303 (25.61)	311 (25.85)	
30–39	250 (21.13)	240 (19.95)	
40–49	252 (21.30)	252 (20.95)	
50–59	214 (18.09)	206 (17.12)	
60–69	113 (9.55)	127 (10.56)	
70+	25 (2.11)	52 (4.32)	
Missing	26 (2.20)	15 (1.25)	
Biological sex, *N* (%)			4.48 × 10^–3^ (**)
Female	746 (63.06)	825 (68.58)	
Male	437 (36.94)	378 (31.42)	
Missing	0	0	
Ethnicity, *N* (%)			6.79 × 10^–3^ (**)
White	1045 (88.33)	1020 (84.78)	
Non-White	135 (11.42)	183 (15.21)	
Mixed Race	77 (6.51)	73 (6.07)	
Asian	18 (1.52)	43 (3.57)	
Latin American/Hispanic	7 (0.59)	23 (1.91)	
Arab/Middle Eastern	0	17 (1.41)	
Jewish	16 (1.35)	17 (1.41)	
African/Black/Caribbean	6 (0.51)	9 (0.75)	
Other	11 (0.93)	1(0.08)	
Missing	3 (0.25)	0	
Education, *N* (%)			2.68 × 10^–17^ (***)
No formal qualifications	57 (4.82)	14 (1.16)	
Further vocational qualifications	215 (18.17)	138 (11.47)	
Secondary school/high school	211 (17.84)	171 (14.21)	
University undergraduate	354 (29.92)	354 (29.43)	
University postgraduate	344 (29.08)	523 (43.47)	
Missing	2 (0.17)	3 (0.25)	
Country of residence			2.59 × 10^–5^ (***)
UK	842 (71.17)	759 (63.09)	
USA	120 (10.14)	174 (14.46)	
Germany	31 (2.62)	33 (2.74)	
Australia	33 (2.79)	20 (1.66)	
Other	156 (13.19)	214 (17.79)	
Missing	1 (0.08)	3 (0.25)	
Body mass index (kg/m^2^), mean (SD)	27.07 (7.52)	26.05 (6.22)	2.25 × 10^–3^ (***)
Body mass index, categories, *N* (%)			
Underweight	73 (6.32)	35 (2.96)	
Normal weight	459 (39.71)	608 (51.35)	
Overweight	308 (26.64)	300 (25.34)	
Obese	316 (27.34)	241 (20.36)	
Missing	27 (2.28)	19 (1.58)	
Most frequent smoking, *N* (%)			0.030 (*)
Never	802 (67.79)	776 (64.51)	
Monthly	2 (0.17)	9 (0.75)	
Weekly	25 (2.11)	40 (3.33)	
Daily	354 (29.92)	377 (31.34)	
Missing	0	1 (0.08)	
Current alcohol frequency, *N* (%)			6.54 × 10^–21^ (***)
0 days per week	693 (58.58)	465 (38.65)	
1–2 days per week	300 (25.36)	470 (39.07)	
3–5 days per week	119 (10.06)	183 (15.21)	
6–7 days per week	70 (5.92)	84 (6.98)	
Missing	1 (0.08)	1 (0.08)	
Intellectual disability (ID), *N* (%)			5.40 × 10^–4^ (***)
Self-identified	21 (1.78)	4 (0.33)	

Significance level: *** (*p* < 0.001), ** (*p* < 0.01), * (*p* < 0.05), ▲ (*p* < 0.10)

*SD *standard deviation

*p* values were from Pearson’s Chi-square test (categorical) or from a Mann–Whitney U test (means)

These are demographic data before imputation. The results remain highly similar after imputation

Further information on recruitment, exclusions, and missingness/non-response is available in Addition file 2.

### Covariates

Education level was defined as the highest qualification achieved with the following options: “No formal qualifications,” “Secondary School/High School level qualifications,” “Further vocational qualifications,” “University Undergraduate level qualifications (BA, BSc, etc.),” and “University Postgraduate level qualifications (MA, MSc, PhD, Certificate, etc.),” as a categorical variable. We used a binary representation of ethnicity (white vs. non-white) in our analyses, as we had very few individuals from each non-white ethnic background. Full information on the distribution of participants’ ethnicity is available in Table 1. We derived a variable from country of residence based on the most frequent countries listed with the following options: “UK,” “USA,” “Germany,” “Australia,” and “Other.” We used frequency of current alcohol consumption, as measured by the number of days per week with the following options: “I do not consume alcoholic beverages,” “One to two days per week,” “Three to four days per week,” and “Five to seven days per week,” as an ordinal variable. Finally, we used highest frequency of smoking ever, as measured by the regularity of smoking when smoking most frequently with the following options: “I have never smoked regularly,” “Monthly,” “Weekly,” and “Daily,” to quantify smoking among participants.

### Statistical analyses

We used *R Version 3.6.2,* and specifically the “CrossTable” function from the “gmodels” package for two-tailed Fisher’s exact tests; and the “wilcox.test,” and “glm” functions from the “stats” package for Wilcoxon signed rank tests, and binomial logistic regression analyses, respectively. We used both unadjusted (Fisher’s) and adjusted models (logistic regression) to assess diet, exercise, and sleep patterns of autistic adults; mediation of epilepsy/seizure disorders on sleep disturbances and sleep duration; differences in BMI (weight classes); relationships between diet, exercise, sleep, and family history with cardiovascular and diabetic outcomes; and sex differences across all analyses.

**Fig. 1:**
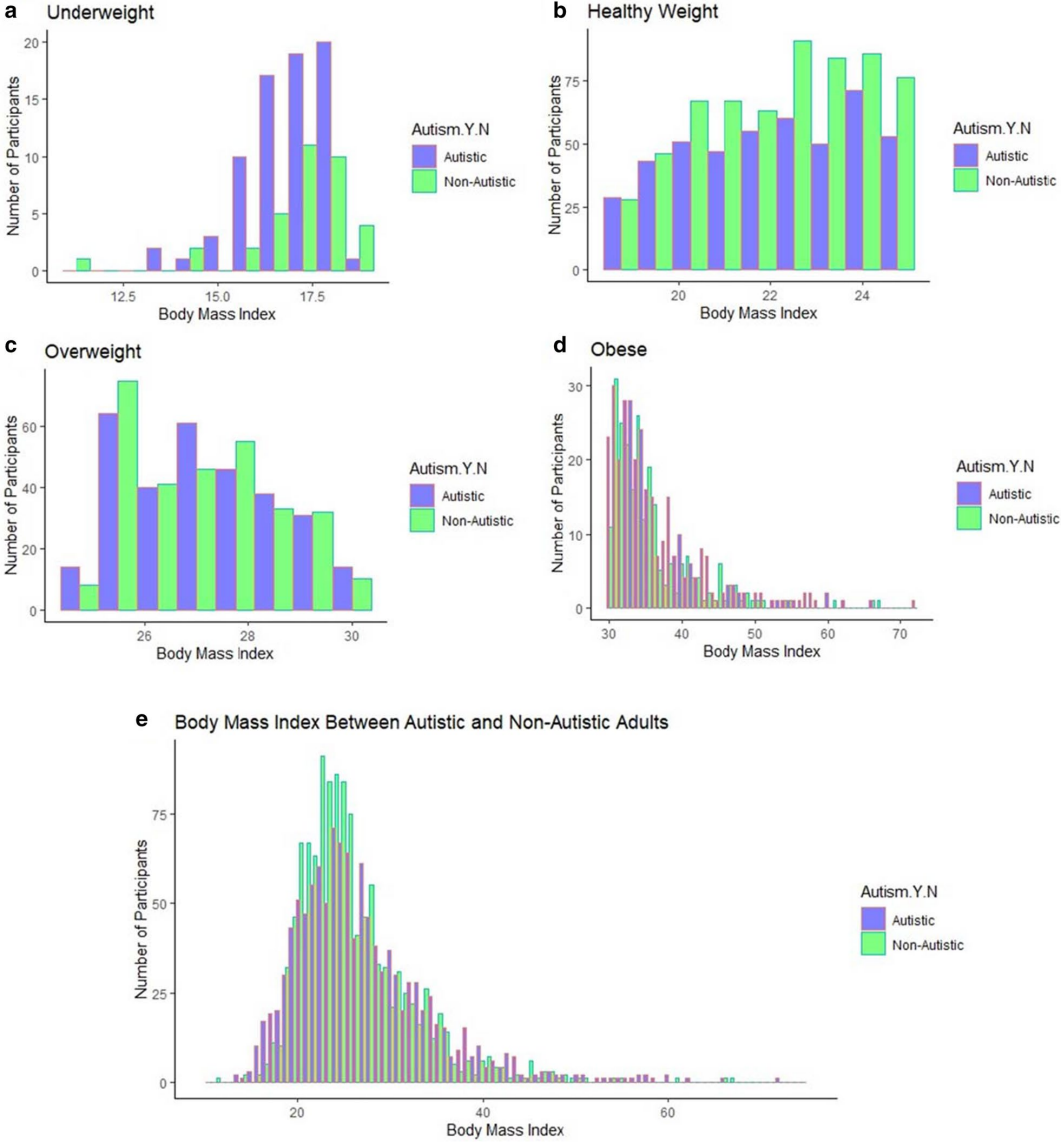
Distribution of BMI among autistic and non-autistic adults. **a–d** The specific distributions of autistic and non-autistic individuals classified as underweight (**a**), normal range (**b**), overweight (**c**), or obese (**d**), and **e** display the full distribution of BMIs for both autistic and non-autistic individuals, based on their self-reported heights and weights

First, we established whether there were differences on average between autistic and non-autistic adults using derived, binary measures: exercising at least once per week, exercising 75 min or more per week, sleeping at least six hours per night, specific sleep disturbances (including difficulty falling asleep, difficulty staying asleep, sleepwalking, sleep talking, bedwetting, frequent night terrors, excessive drowsiness, narcolepsy, and sleep apnea), eating at least five servings of fruit or vegetables on four or more days per week, eating high calorie foods frequently (seven or more times per week), meeting water goals (at least seven glasses/cups of water per day), drinking high sugar beverages frequently (seven or more times per week), drinking caffeinated beverages frequently (seven or more times per week), reporting specific dietary restrictions (including vegan or vegetarian, lactose free, nut free, gluten free, soy free, no fish, other dietary restriction), reporting no dietary restriction, and reporting dietary restriction due to an allergy. As noted above, we used both unadjusted and adjusted models (controlling for biological sex, age, ethnicity, education level, and country of residence). For full details on these items, see Addition file 1: Figs. 1–11.

### Analysis of differences in BMI (weight classes)

We derived BMI (kg/m^2^) and weight classes based on guidance given by the WHO (https://www.who.int/dietphysicalactivity/childhood_what/en/) and tested differences between autistic and non-autistic individuals in the prevalence of being classified as underweight (BMI of < 18.5), normal range weight (BMI of ≥ 18.5 but < 25.0), overweight (BMI ≥ 25.0 but < 30.0), or obese (BMI of ≥ 30.0). In model two, we used binomial logistic regression and controlled for demographic factors including sex, age, ethnicity, education level, and country of residence, as well as social factors including current alcohol use, and past smoking frequency. In model three, we again used binomial logistic regression and controlled for the same demographic and social history factors, as well as ordinal measures of a variety of lifestyle factors that we expect would impact BMI: exercise frequency, exercise duration, sleep duration, meeting fruit and vegetable goals, eating high calorie foods, drinking high sugar beverages, meeting water goals, drinking caffeinated beverages, sleep disturbance (yes/no), and dietary restriction (yes/no). We ran sensitivity analyses to consider the effects of an interaction between sex and diagnosis on each of the specific weight classes (underweight, normal range, overweight, and obese) for both models two and three and found no significant interactions.

### Analysis of health: cardiovascular and diabetic outcomes (sex-stratified)

As mentioned above, we collected medical history data for common non-communicable diseases, including data on lifetime prevalence of cardiovascular and diabetic conditions. For these analyses, we derived two binary variables to indicate whether each individual self-reported any non-communicable cardiovascular condition or separately any non-communicable diabetic condition. For example, an individual who reported high blood pressure would be designated as having a cardiovascular condition. See Addition file 1: Figs. 8 and 9 for further information about the specific cardiovascular and diabetic conditions considered. Similarly, we used self-report data on first-degree biological relatives to derive a binary “Family History” variable to indicate whether participants reported that a family member had any non-communicable cardiovascular or diabetic condition, respectively. As prevalence of health outcomes may depend on biological sex [3, 4, 7], these analyses compared rates of medical conditions among autistic females vs. non-autistic females, and separately, autistic males vs. non-autistic males.

We employed four statistical models to examine the effects of demographics, social history, lifestyle, and family history on risk of cardiovascular and diabetic outcomes between sex-matched groups. We used Fisher’s exact tests for model one and used binomial logistic regression for models two, three, and four. As above, we controlled for demographic and social history factors in model two and demographic, social history factors, and family history in model three. Finally, in model four, we controlled for the same demographic and social history factors, as well as lifestyle factors (specific factors listed above under the *Analysis of Differences in BMI (Weight Classes)* subsection).

We have previously reported similar results to that of model one and model two for sex-stratified analyses of cardiovascular and diabetic conditions using data from the same sample [7]. We included these analyses in order to provide clear points of comparison for models three and four (controlling for lifestyle factors and family history, respectively), which have not been investigated or reported previously.

### Sex differences across all analyses

As noted in the subsections above, we assessed sex differences throughout the study. We conducted additional analyses using binomial logistic regression controlling for age, biological sex, ethnicity, education, country of residence, and the interaction of sex and diagnosis. Where we found a significant interaction between sex and diagnosis, we employed the “glht” function of the “multcomp” package to estimate sex-specific information, where appropriate, we reported sex-specific results in place of the main models in Table 2.

**Table 2:** Exercise, diet, and sleep patterns of autistic adults compared to non-autistic adults

	Unadjusted model		Adjusted model†		
	OR (95% CI)	FDR	OR (95% CI)	FDR	Sig
Exercising at least once per week	0.641 (0.536, 0.766)	1.96 × 10^–6^	0.690 (0.576, 0.826)	1.62 × 10^–4^	***
Meeting weekly exercise goals	0.593 (0.491, 0.715)	1.07 × 10^–7^	0.629 (0.520, 0.760)	6.40 × 10^–6^	***
Meeting fruit and vegetable goals (females)	0.550 (0.448, 0.676)	3.35 × 10^–8^	0.596 (0.441, 0.805)	1.93 × 10^–3^	**
Meeting fruit and vegetable goals (males)	1.075 (0.808, 1.430)	0.623	1.086 (0.714, 1.652)	1.000	
Eating high calorie foods	1.251 (1.042, 1.502)	0.022	1.240 (1.031, 1.491)	0.030	*
Meeting daily water goals	1.073 (0.884, 1.302)	0.504	1.195 (0.980, 1.457)	0.091	
Drinking high sugar beverages	1.492 (1.103, 2.027)	0.011	1.328 (0.978, 1.802)	0.083	
Drinking caffeinated beverages	0.728 (0.612, 0.865)	4.79 × 10^–4^	0.764 (0.637, 0.917)	5.90 × 10^–3^	**
Vegan/vegetarian	1.303 (1.043, 1.631)	0.023	1.401 (1.118, 1.755)	5.36 × 10^–3^	**
Lactose free	2.152 (1.579, 2.954)	1.48 × 10^–6^	2.257 (1.655, 3.076)	1.67 × 10^–6^	***
Nut free	1.514 (0.837, 2.790)	0.186	1.748 (0.982, 3.112)	0.075	
Gluten free	2.375 (1.702, 3.346)	3.25 × 10^–7^	2.436 (1.744, 3.402)	1.42 × 10^–6^	***
Soy free	1.771 (0.954, 3.382)	0.073	1.768 (0.965, 3.240)	0.081	
No fish	1.772 (1.152, 2.760)	0.011	1.905 (1.246, 2.912)	4.91 × 10^–3^	**
Other dietary restriction	1.663 (1.338, 2.071)	7.68 × 10^–6^	1.758 (1.410, 2.193)	2.97 × 10^–6^	***
No dietary restriction	0.740 (0.627, 0.872)	5.32 × 10^–4^	0.690 (0.584, 0.817)	5.31 × 10^–5^	***
Dietary restriction due to allergy	1.856 (1.419, 2.435)	7.99 × 10^–6^	1.953 (1.491, 2.558)	5.58 × 10^–6^	***
Sleeping 6+ hours (females)	0.617 (0.489, 0.777)	6.63 × 10^–5^	0.630 (0.451, 0.882)	2.22 × 10^–3^	**
Sleeping 6+ hours (males)	0.905 (0.640, 1.277)	0.623	1.029 (0.627, 1.690)	1.000	
Difficulty falling asleep (females)	2.140 (1.694, 2.710)	3.23 × 10^–10^	1.997 (1.427, 2.796)	1.93 × 10^–3^	**
Difficulty falling asleep (males)	1.399 (1.023, 1.915)	0.039	1.293 (0.825, 2.027)	0.744	
Difficulty staying asleep	1.979 (1.666, 2.354)	1.85 × 10^–14^	2.108 (1.765, 2.517)	2.22 × 10^–15^	***
Sleepwalking	1.530 (1.193, 1.968)	1.15 × 10^–3^	1.542 (1.202, 1.979)	1.79 × 10^–3^	**
Sleep talking	1.295 (1.079, 1.555)	7.66 × 10^–3^	1.288 (1.071, 1.550)	0.010	*
Bedwetting (females)	2.166 (1.516, 3.120)	2.26 × 10^–5^	2.039 (1.229, 3.383)	1.93 × 10^–3^	**
Bedwetting (males)	1.316 (0.861, 2.026)	0.243	1.169 (0.637, 2.147)	1.000	
Frequent night terrors^††^	3.248 (2.536, 4.185)	6.82 × 10^–22^	3.109 (2.424, 3.989)	2.22 × 10^–15^	***
Excessive drowsiness^††^	2.290 (1.930, 2.720)	2.78 × 10^–21^	2.179 (1.833, 2.590)	2.22 × 10^–15^	***
Narcolepsy	2.980 (1.346, 7.260)	6.08 × 10^–3^	3.460 (1.576, 7.595)	3.72 × 10^–3^	**
Sleep apnea	1.578 (1.146, 2.183)	6.75 × 10^–3^	1.645 (1.187, 2.280)	4.91 × 10^–3^	**

^†^The adjusted model typically refers to binomial logistic regression adjusting for age, biological sex, ethnicity, education, and country of residence. In a sensitivity analysis controlling for interaction of sex and diagnosis, if we found a significant interaction, we have reported sex-specific values from binomial logistic regression adjusting for age, biological sex, interaction of biological sex and diagnosis, ethnicity, education, and country of residence

^††^ Exact p-value was < 2.22 × 10^–16^ and could not be precisely determined; for the purposes of the FDR calculations, we estimated this p-value as equal to 2.22 × 10^–16^, in order to provide the most conservative analysis

Significance level: *** (p < 0.001), ** (p < 0.01), * (p < 0.05), ▲ (p < 0.10)

OR = odds ratio; 95% CI = 95% confidence interval; FDR = false discovery rate; Sig. = significance level

## Results

Even after accounting for biological sex, age, ethnicity, education level, and country of residence, autistic individuals were less likely to meet very minimal health recommendations for diet, exercise, and sleep on nearly all measures tested. Our adjusted regression analyses suggest that for every 10 non-autistic adults that report exercising once per week or for at least 75 min per week, only 6–7 autistic adults report the same. Autistic adults were more likely than non-autistic adults to eat high calorie foods frequently; they were also more likely than non-autistic adults to have every dietary restriction (odds ratios: 1.40–2.44) and sleep disturbance (odds ratios: 1.29–3.46) tested. We found marginally significant differences in the likelihood of meeting daily water goals, drinking high sugar beverages frequently, as well as being nut free or soy free.

There were some significant sex differences of note. Autistic females were less likely to eat five servings of fruits and vegetables on at least four days per week or sleep six hours per night than sex-matched peers; they were more likely than non-autistic females to experience difficulty falling asleep and bedwetting. Interestingly, there were no differences between autistic and non-autistic male groups on these measures. Full results for both unadjusted and adjusted models are summarized in Table 2.

As some studies in children suggest that child seizures/epilepsy may contribute to sleep disturbances and sleep duration [20], we conducted additional regression analyses to test this in an adult population (Addition file 2: Table 2). Interestingly, we found no statistically significant effect from epilepsy or seizure disorders on the likelihood of sleeping six hours per night or incidence of sleep disturbances examined here.

Our results confirm previous findings that autistic individuals are more likely than non-autistic adults to be underweight or obese, as well as less likely to be a healthy weight for all three models. However, we found no statistical differences between the likelihood of autistic and non-autistic individuals being overweight. In addition, lifestyle factors do not explain differences in BMI and only contribute to an attenuation of odds ratios for underweight individuals. Full details are provided in Table 3. Figure 1 also includes the unadjusted BMI for autistic and non-autistic individuals.

**Table 3:** Differences in the likelihood of being classified as underweight, overweight, or obese among autistic and non-autistic adults

	Model 1		Model 2		Model 3	
	Unadjusted		Adjusted for potential confounders†		Adjusted for confounders and lifestyle factors††	
	OR (95% CI)	FDR (Sig.)	OR (95% CI)	FDR (Sig.)	OR (95% CI)	FDR (Sig.)
Underweight	2.212 (1.445, 3.441)	1.45 × 10^–4^ (***)	2.308 (1.493, 3.568)	3.40 × 10^–4^ (***)	2.050 (1.309, 3.210)	3.46 × 10^–3^ (**)
Normal Range	0.624 (0.528, 0.737)	6.46 × 10^–8^ (***)	0.644 (0.542, 0.765)	2.28 × 10^–6^ (***)	0.676 (0.565, 0.809)	8.29 × 10^–5^ (***)
Overweight	1.070 (0.886, 1.293)	0.480	1.095 (0.901, 1.331)	0.361	1.081 (0.883, 1.324)	0.448
Obese	1.472 (1.210, 1.792)	1.45 × 10^–4^ (***)	1.376 (1.126, 1.682)	2.43 × 10^–3^ (**)	1.335 (1.079, 1.651)	0.011 (*)

Significance level: *** (p < 0.001), ** (p < 0.01), * (p < 0.05), ▲ (p < 0.10)

OR = odds ratio; 95% CI = 95% confidence interval; FDR = false discovery rate; Sig. = significance level

^†^ Binomial logistic regression adjusting for sex, age, ethnicity, education level, country of residence, current alcohol use, and past smoking frequency

^††^ Binomial logistic regression adjusting for sex, age, ethnicity, education level, country of residence, current alcohol use, past smoking frequency, exercise frequency, exercise duration, sleep duration, meeting fruit and vegetable goals, eating high calorie foods, drinking high sugar beverages, meeting water goals, drinking caffeinated beverages, sleep disturbance (yes/no), and dietary restriction (yes/no)

Previous studies suggest that autistic adults are more likely to have cardiovascular and diabetic conditions [3, 4, 7]; however, this study is the first to consider the relationships between lifestyle-related factors, family history, and chronic disease among autistic adults. Interestingly, after controlling for lifestyle-related factors (comparing Model 2 to Model 4), risk of cardiovascular conditions drops from odds ratios of 1.53 to 1.41 and there is no longer a significant difference between autistic males and non-autistic males—suggesting that lifestyle factors may be associated with increased risk of cardiovascular diseases. Surprisingly, when controlling for these same lifestyle-related factors, the relative risk of diabetic conditions among autistic females increases. Finally, in contrast to previous findings [3, 5], our study does not provide strong evidence that there are true group differences between autistic and non-autistic males regarding risk of diabetic conditions, as only Model 1 suggests a significant difference. Full results for all four models are provided in Table 4.

**Table 4:** The effect of lifestyle factors on risk of cardiovascular and diabetic outcomes between autistic and non-autistic adults, stratified by sex

	Model 1		Model 2		Model 3		Model 4	
	Unadjusted		Adjusting for potential confounders†		Adjusting for potential confounders and family history††		Adjusting for potential confounders and lifestyle factors†††	
	OR (95% CI)	FDR (Sig.)	OR (95% CI)	FDR (Sig.)	OR (95% CI)	FDR (Sig.)	OR (95% CI)	FDR (Sig.)
Female cardiovascular conditions	1.291 (1.142, 1.459)	4.88 × 10^–5^ (***)	1.442 (1.070, 1.943)	0.033 (*)	1.444 (1.067, 1.954)	0.034 (*)	1.438 (1.037, 1.995)	0.059 ( ▲)
Male cardiovascular conditions	1.407 (1.207, 1.641)	1.69 × 10^–5^ (***)	1.525 (1.024, 2.270)	0.050 **(** ▲**)**	1.506 (1.010, 2.245)	0.059 **(** ▲**)**	1.413 (0.913, 2.188)	0.161
Female Diabetic Conditions	1.526 (1.274, 1.830)	1.01 × 10^–5^ (***)	1.948 (1.261, 3.008)	0.011 (*)	1.870 (1.206, 2.898)	0.021 (*)	2.315 (1.419, 3.778)	3.18 × 10^–3^ (**)
Male Diabetic Conditions	1.671 (1.271, 2.207)	1.39 × 10^–4^ (***)	1.663 (0.852, 2.243)	0.136	1.678 (0.859, 3.278)	0.130	1.537 (0.723, 3.267)	0.264

Significance level: *** (p < 0.001), ** (p < 0.01), * (p < 0.05), ▲ (p < 0.10)

OR = odds ratio; 95% CI = 95% confidence interval; FDR = false discovery rate; Sig. = significance level

^†^ Binomial logistic regression adjusting for age, ethnicity, education level, country of residence, current alcohol use, and past smoking frequency

^††^ Binomial logistic regression adjusting for age, ethnicity, education level, country of residence, current alcohol use, past smoking frequency, and binary measure of family history

^†††^ Binomial logistic regression adjusting for age, ethnicity, education level, country of residence, current alcohol use, past smoking frequency, exercise frequency, exercise duration, sleep duration, meeting fruit and vegetable goals, eating high calorie foods, drinking high sugar beverages, meeting water goals, drinking caffeinated beverages, sleep disturbance (yes/no), and dietary restriction (yes/no)

## Discussion

Our results extend previous findings from children to suggest that autistic adults are less likely to follow even minimal health recommendations for diet, exercise, and sleep, which may affect wider quality of life. A recent latent class analysis of autistic children found relationships between health, weight, engagement in school, ability to make friends, physical activity, sleep habits, and screen time [73]. In addition, autistic children were more likely to be in the classes with negative behaviors and outcomes and far less likely to be in the classes with positive behaviors and outcomes [73]. Further, sleep problems have previously been associated with daytime sedentary behavior and unemployment among autistic adults [70, 74] and with increased mental health difficulties among those who self-report high autistic traits [75]; however, there is no existing literature that considers the effects of diet, exercise, and sleep habits broadly on the quality of life of autistic adults. Considering the widespread challenges faced by autistic adults, failure to meet minimum recommendations for diet, exercise, and sleep could have knock-on effects in a variety of different areas including but not limited to: increasing physical health risks; limiting opportunities for social engagement (which can often be centered around mealtimes or physical activity); worsening the severity of behavioral problems and/or comorbid psychiatric conditions; and increasing likelihood of unemployment, underemployment, or attrition from education. Thus, future research in this area should focus on exploring the possible impacts that lifestyle choices might have on the quality of life of autistic adults.

Our online recruitment methods allowed us to reach a large cohort of autistic individuals across the adult lifespan (aged 16–90 years; mean age approximately 41 years). This is the largest study of its kind and provides perspectives from middle-aged and older autistic adults, who remain a neglected group in autism research. In addition, this is the first study to describe the dietary choices and restrictions of autistic adults in a large population of individuals without ID. The study also includes a large sample of autistic females, who remain underserved in research and clinical practice, despite further increased risks of chronic health conditions and premature mortality (even in comparison with autistic males) [3, 5, 7, 9]. Autistic females may be particularly vulnerable to challenges with sleeping at least six hours per night, difficulty falling asleep, bedwetting, and likelihood of meeting fruit and vegetable goals at least four days per week compared to sex-matched peers. Our sample predominantly comprised females, which was expected based on methodology of using an online, self-report survey [76–79]. This has likely biased the sample toward the perspectives and experiences of females and in turn will limit its applicability and power in detecting true group differences between autistic and non-autistic males; however, our study fits into a wider narrative suggesting increased challenges and inadequate support for autistic individuals—and particularly autistic females [3–5, 7, 9, 80, 81]. Future qualitative research should attempt to establish what types of support would be most effective in trying to improve daily living and functional outcomes for autistic individuals and autistic females specifically.

It has also been suggested that there may be a relationship between epilepsy/seizure disorders, sleep duration, and frequency of sleep disturbances [65]. Yet, our results indicate that at least some autistic individuals experience sleep disturbances and reduced sleep duration independently of epilepsy/seizure disorders. It should be noted that our methodology, by definition, requires that all participants be intellectually and physically able to fill in a self-report survey; it is possible that epilepsy/seizure disorders may play a larger role in sleep duration and sleep disturbances of individuals with greater autism severity and/or ID.

Across all three models, we found that autistic individuals were more likely to be classified as underweight or obese, as well as less likely to be classified as having a normal range BMI. After accounting for factors related to diet, sleep, and exercise, there was a modest attenuation in odds ratios and the significance level for underweight individuals, indicating that lifestyle factors may contribute to some aspects of atypical BMI; however, a statistically significant difference remained between autistic and non-autistic individuals in regard to the likelihood of being classified as underweight, normal range, or obese for BMI. Thus, our results intimate that lifestyle patterns do not fully explain the differences in BMI observed between autistic and non-autistic adults in this sample; however, it is presently unclear whether these persistent differences in BMI can be explained by biases from our sample (detailed below) or other differences common to autism (e.g., genetic mutations, hormonal dysregulation, etc.) [82–85].

Our results provide the first evidence that unhealthy diet, exercise, and sleep patterns may appreciably relate to the excess risks of cardiovascular conditions seen among autistic adults compared to non-autistic adults [3–5, 7], though our methodology is not able to test causality. This is represented by a pattern of attenuation in significance and odds ratios seen uniquely in Model 4 for autistic males. Remarkably, this pattern of attenuation is stronger when accounting for the cumulative effects of lifestyle-related factors even than for family history of a cardiovascular condition. A similar pattern of attenuation in significance is shown among autistic females; however, we did not observe a similar effect on the odds ratios. Thus, this study cannot yet provide evidence of such an effect among autistic females. In addition to providing key information regarding excess health risks, this finding may have wider implications for length and quality of life, as studies suggest that increased health burden contributes to premature death among autistic adults [8, 9]. Future research must work to establish these relationships and identify whether lifestyle behaviors could serve as key targets for intervention for improving long-term outcomes among autistic adults.

Our results also indicate that unhealthy lifestyle-related factors may be related to lower risk of diabetic conditions among autistic females (as odds ratios and significance increase when covarying for diet, exercise, and sleep patterns). While it appears true that these lifestyle factors still affect likelihood of developing diabetic conditions, our results suggest that other factors may more meaningfully contribute to these outcomes, such as genetic mutations [82], hormonal conditions [83–85], negative life experiences [86], mental health conditions [3–5, 87], and lack of access to healthcare and/or poor quality of healthcare [80, 81]. In particular, this study provided some preliminary evidence that family history of diabetic conditions may contribute more to increased risk of diabetic conditions among autistic females than lifestyle-related factors. As we cannot test causality, the present study cannot determine whether this unexpected pattern is based on true group differences or indicative of reverse causality: autistic females are more likely to make lifestyle changes based on a diagnosis of diabetes or prediabetes.

These findings underscore the importance of timely autism diagnosis, as patterns of unhealthy lifestyle behaviors appear to persist from childhood into adulthood. Although future research is needed, our study provides preliminary, correlational evidence that diet, exercise, and sleep should be investigated further as possible contributors to uniquely increased risks of health burden and premature death among autistic individuals [3–9]. Thus, physicians should work cooperatively with their autistic patients (and particularly autistic females) to improve health education; monitor challenges with diet, sleep, and exercise; and develop strategies to provide support, in order to reduce risks. Future qualitative research should focus on identifying the causes of these differences (e.g., lack of health education, biological reasons for disruptions, mental health conditions making it harder to manage) and creating mechanisms to provide better support to the autistic community.

## Limitations

Our study employed a self-report measure; thus, it relied on participants to provide an accurate account of their diet, exercise, and sleep patterns, as well as weight, height, physical health history, and family history. Further, social history and lifestyle patterns of autistic individuals are complex and difficult to distill effectively in a few questions; as such, our study only accounts for participants’ current alcohol consumption frequency and the frequency of their smoking when smoking the most, which may not fully capture alcohol and smoking behaviors. Although the survey was anonymized, autistic individuals may have been more candid about their experiences and unhealthy behaviors, due to differences in communication style and/or lessened concerns about adherence to social norms; however, it should be noted that our results align with studies of autistic individuals across all ages, including studies utilizing parent report measures.

The study may also be subject to sampling bias, as we recruited participants via various sources of social media, autism support groups, and autism charities. Although we encouraged participation from both autistic and non-autistic individuals, our advertisement methods may have biased our control population toward individuals with an interest in autism or who suspect they are autistic. We excluded all individuals that reported self-diagnosis of autism, suspected autism, or were awaiting an autism assessment; nevertheless, our results may underestimate true group differences between autistic and non-autistic individuals.

In addition, our study sample may not be representative of all autistic individuals. First, our methodology inherently excluded individuals without internet access. Second, less than 2% of our sample self-identified as having an intellectual, developmental, cognitive, or learning disability (not including specific learning disabilities). Current research into lifestyle-related patterns among autistic adults is heavily biased toward autistic individuals with comorbid ID (particularly regarding diet, as there are no other large-scale studies describing dietary restrictions or quality among autistic adults without ID); yet, it is important to note that the results of this study may not be generalizable to the entire autistic population. Third, although we attempted to recruit a diverse and international sample, several groups (e.g., females, UK residents, individuals with at least a high school education, and individuals of a White/Caucasian ethnicity) were overrepresented in both the autistic and non-autistic groups. Thus, these findings may not be representative of the experiences of all autistic or non-autistic adults and particularly of autistic males. The present study may be underpowered to detect all sex differences and may not accurately represent the experiences or health risks of non-white autistic individuals or those with lower educational achievement. It is possible that this study underestimates true group differences in diet, exercise, sleep, BMI, and health outcomes for diverse and underserved populations, as additional intersections of disability, ethnicity, and socioeconomic status may serve to magnify the effects seen here. Future research must focus on filling these gaps by actively recruiting ethnically diverse autistic individuals, who remain an underserved population in research.

Further, our cross-sectional design limits the conclusions we can draw, and specifically our ability to test the direction of relationships and/or causality. As noted above, we found an inverse relationship between diabetic conditions and lifestyle choices. It may be the case that other, aforementioned biological and environmental factors better explain the excess risk of diabetic conditions among autistic females than do lifestyle factors. Alternatively, our results may be explained by reverse causality. As this study only includes data from one timepoint, future, longitudinal studies should focus on clarifying the relationship between lifestyle choices and risk of diabetic conditions, as well as identifying the biological and environmental factors which contribute most meaningfully to this risk among autistic females.

## Conclusions

Overall, despite lower prevalence of smoking and alcohol use, autistic adults are less likely to meet minimal health recommendations for diet, exercise, and sleep than are non-autistic adults, and this may be particularly true for autistic females (compared to sex-matched peers). In particular, our findings provide correlational evidence that unhealthy habits may appreciably relate to excess risk of cardiovascular conditions seen among autistic males (though we cannot test causality). Autistic adults are also more likely to be classified as underweight or obese and less likely to be classified as within the normal weight range regarding BMI; interestingly, atypical BMI is not fully explained by diet, exercise, or sleep patterns. It should be noted that these results may not apply to all autistic individuals and may primarily apply to white, female, UK residents, and those who have completed at least a high school education. However, the present study emphasizes the urgency of developing strategies to better support autistic individuals in making healthy lifestyle choices.

## Supplementary Information

**Additional file 1.** Supplemental Figures 1–11.

**Additional file 2.** Supplementary Information.

## Data Availability

As participants did not consent for their data to be publicly shared, even anonymized, data will be made available to only potential collaborators with ethical approval after they submit a research proposal to the Autism Research Centre, University of Cambridge, UK.

## Abbreviations

BMI: Body Mass Index
ID: Intellectual Disability
ARFID: Avoidant and Restricted Food Intake Disorder
IQ: Intellectual Quotient
AQ-10: Autism Quotient (10 Item shortened version)
