# Supplementary material for: An investigation of the diet, exercise, sleep, BMI, and health outcomes of autistic adults
Source: Mol Autism. 2021 May 8;12:31. doi: 10.1186/s13229-021-00441-x (PMC8106173; doi:10.1186/s13229-021-00441-x)

**Supplementary Figures 1-11**

***Supplementary Figure 1
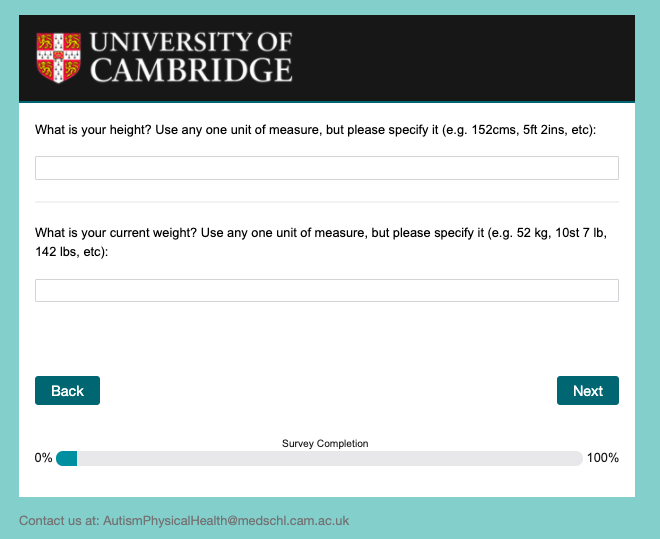
***

***Supplementary Figure 2***

***
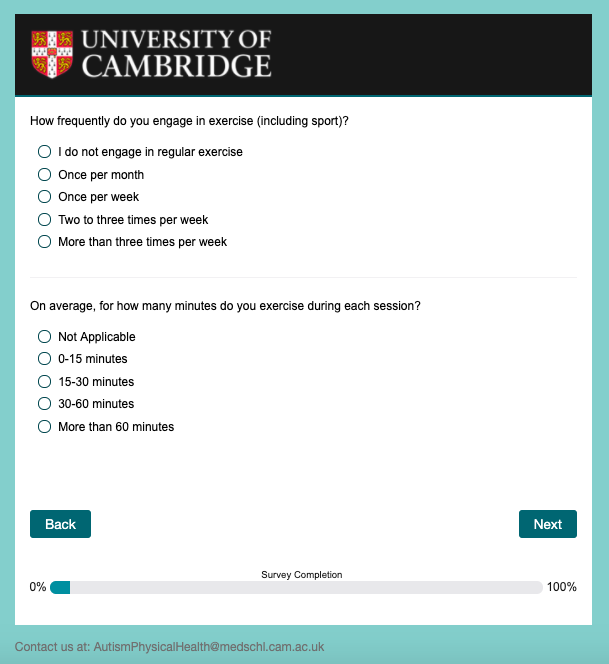
***

***Supplementary Figure 3***

***
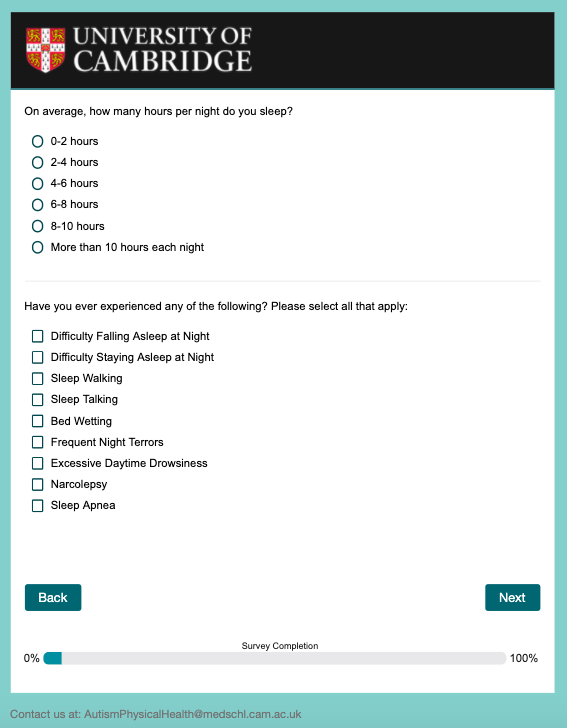
***

***Supplementary Figure 4***

***
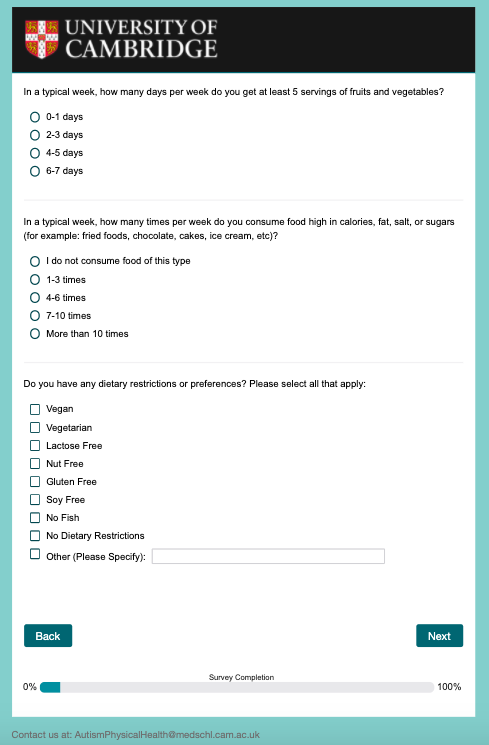
***

***Supplementary Figure 5***

***
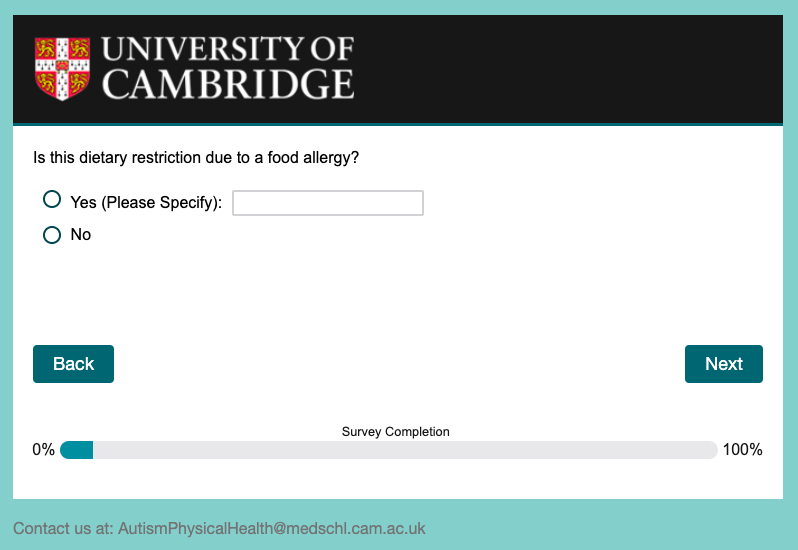
***

***Supplementary Figure 6***


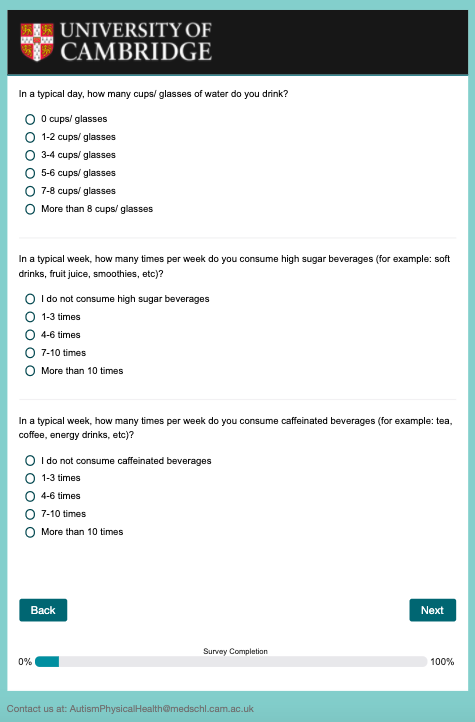


***Supplementary Figure 7***


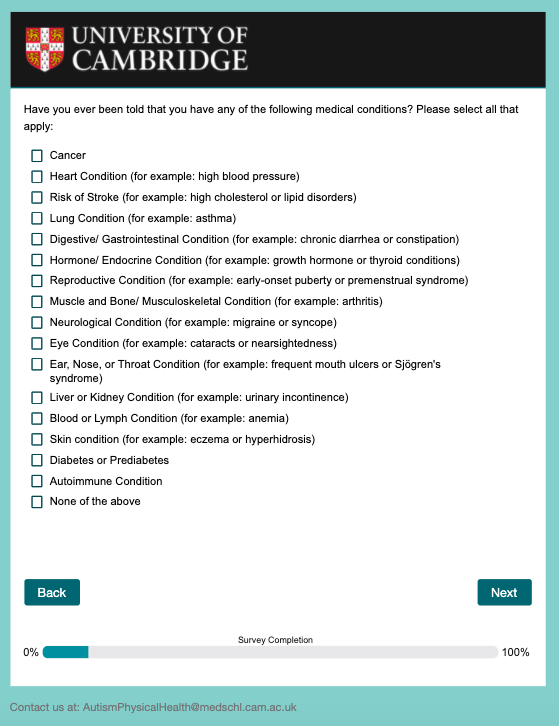


***Supplementary Figure 8***

***
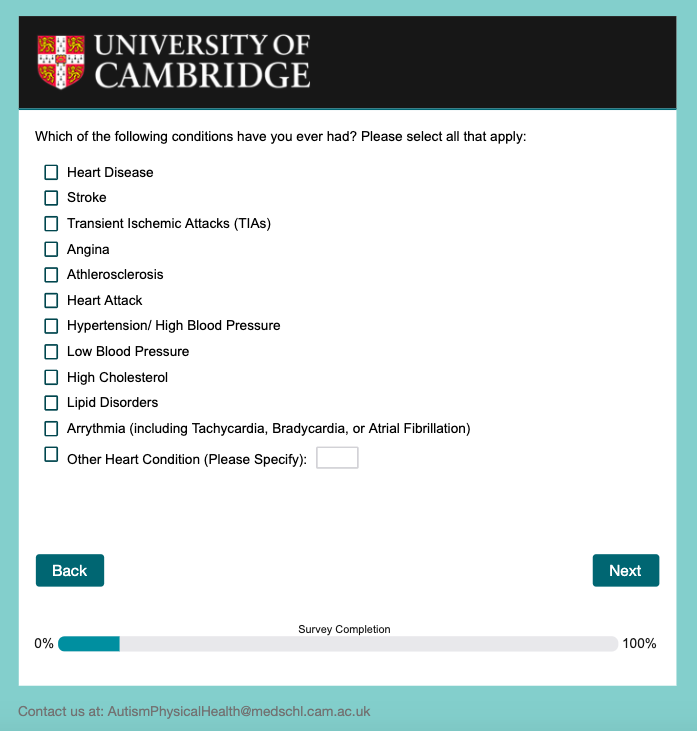
***

***Supplementary Figure 9***


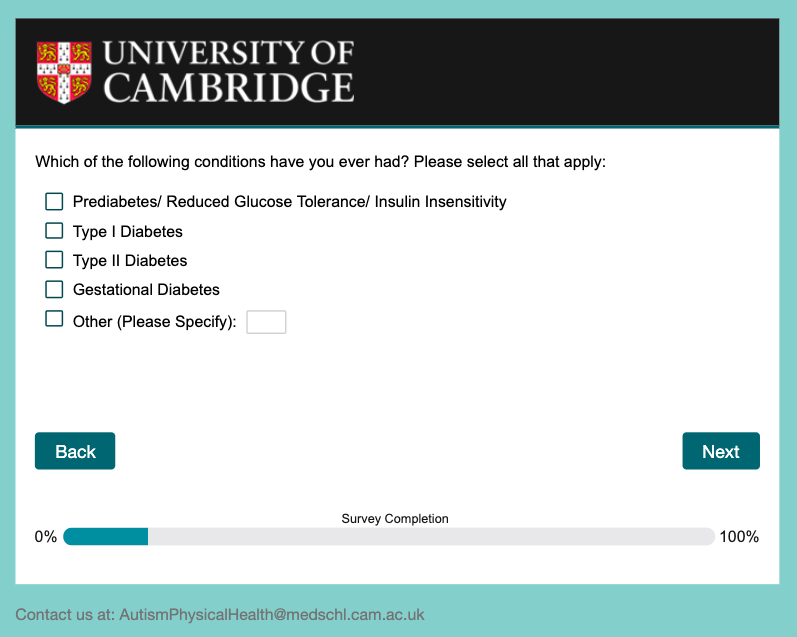


***Supplementary Figure 10***


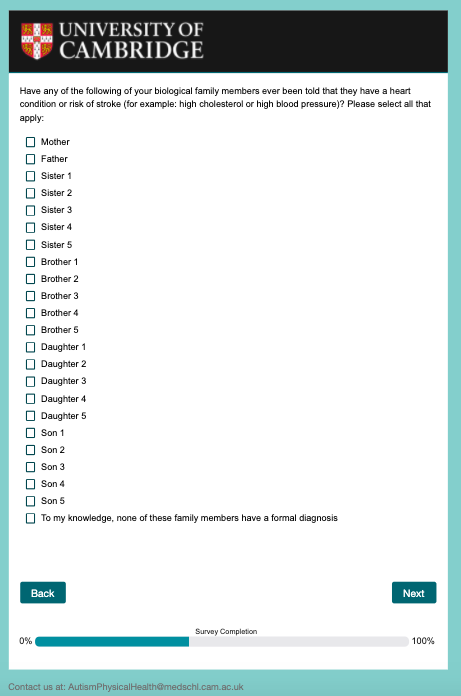


***Supplementary Figure 11***


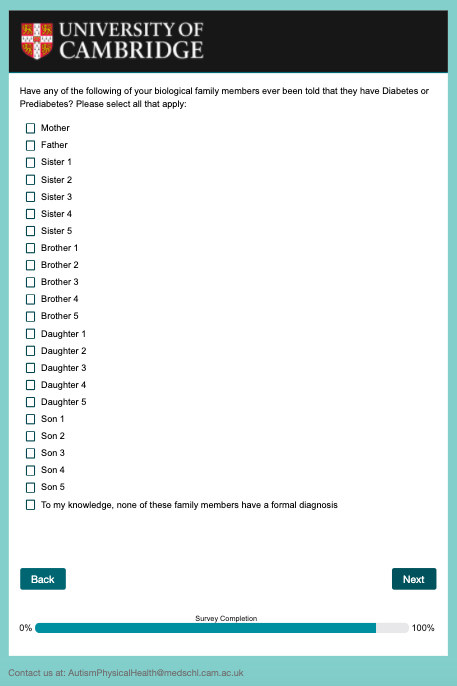

Supplement: Supplementary file 1 — Additional file 1. Supplemental Figures 1–11. [file 13229_2021_441_MOESM1_ESM.docx]
