# Supplementary material for: An investigation of the diet, exercise, sleep, BMI, and health outcomes of autistic adults
Source: Mol Autism. 2021 May 8;12:31. doi: 10.1186/s13229-021-00441-x (PMC8106173; doi:10.1186/s13229-021-00441-x)
Supplement: Supplementary file 2 — Additional file 2. Supplementary Information. [file 13229_2021_441_MOESM2_ESM.docx]

**Supplementary Information**

**Methodology:**

*Recruitment*

This cross-sectional study utilized an online, convenience sampling framework, and recruited participants via the Cambridge Autism Research Database (CARD), Autistica’s Discover Network, autism support groups and charities (including the Autism Research Trust), and social media (specifically Twitter and Facebook). As we advertised to some groups/ forums related to autism, these sampling methods may be bias our control group toward individuals with high autistic traits and/or those with an interest in autism; yet, all advertisements were advertised to and encouraged participation from both autistic and non-autistic individuals. In addition, we used Facebook to advertise our study to the general population and did not target specific autism groups/ forums, in an attempt to limit bias from recruiting individuals via only autism-specific groups/ forums; in this phase of recruitment, both autistic and non-autistic Facebook users from around the world were invited to participate. Although the survey was only available in English, the study aimed to include an international cohort of individuals, and respondents from 62 different countries were included in the sample.

Survey collection took place between February 2018 and August 2019. There were two periods during this time where survey collection was paused to assess new advertisement strategies; there were no changes made to the survey during either of these periods. In sensitivity analyses, we found no statistically significant covariation in any variable by time period (Supplementary Table 3).

*The Cohort*

N = 3,657 individuals responded to the survey. Any individual who was at least 16 years of age and consented to participate was eligible for the study. N = 1,102 individuals were excluded from this analysis due to ‘incomplete’ response, meaning that they exited the survey before completing the lifestyle factors section. 914 of the individuals excluded due to incomplete response (83%) failed to complete the demographics section of the survey (and answered no questions related to lifestyle or physical health), making their responses unusable for this analysis. Not all questions were required, and individuals were not excluded from analysis if they chose to skip optional questions. We also excluded one individual that indicated ‘Other’ for their biological sex, as our analysis strategy considers sex differences in lifestyle patterns.

As the survey was anonymous, we developed an algorithm to exclude potential duplicate responses (n = 112). We excluded all records that matched a previous record on 11 criteria (autism diagnosis (yes/no), specific autism diagnosis, type of diagnosing practitioner, year of autism diagnosis, country of residence, biological sex, current gender identity, education-level, age, maternal age at birth, and paternal age at birth).

We used a case-control design to divide our sample into two groups: an autistic and a control cohort. The autistic cohort included consenting individuals aged 16 years or older with autism diagnosed by a medical practitioner. As the survey was anonymous, autism diagnoses were self-reported; however, we asked participants to provide additional information to verify their diagnosis, such as the type of practitioner who diagnosed them (e.g. psychiatrist, clinical psychologist, pediatrician, etc), year of their diagnosis, specific diagnosis (Autism Spectrum Disorder, Asperger’s, etc), and whether they have a syndromic form of autism. The control group included consenting individuals aged 16 years or older who had not received a formal diagnosis of autism. We then excluded any individuals who self-diagnosed as autistic, suspected autism, or were waiting to be assessed for autism from both the autistic and non-autistic (control) groups, in order to preserve a case-control design (n = 56).

*Missingness/ Non-Response*

**Supplementary Table 1: Number of Individuals Included in Each Analysis**

| **Analyses with Missing Outcome Data** | **Autism**  **N included (% of total)** | **Controls**  **N Included (% of total)** |
| --- | --- | --- |
| Descriptive Analyses |  |  |
| Exercise Frequency | 1,182 (99.92) | 1,203 (100) |
| Meeting Weekly Exercise Goals | 1,179 (99.66) | 1,203 (100) |
| Sleep Duration | 1,182 (99.92) | 1,202 (99.92) |
| Meeting Fruit and Vegetable Goals | 1,179 (99.66) | 1,203 (100) |
| Eating High Calorie Foods | 1,182 (99.92) | 1,203 (100) |
| Meeting Daily Water Goals | 1,180 (99.75) | 1,202 (99.92) |
| Drinking High Sugar Beverages | 1,182 (99.92) | 1,200 (99.75) |
| Drinking Caffeinated Beverages | 1,180 (99.75) | 1,203 (100) |
| Dietary Restriction Due to Allergy | 625 (52.83)* | 545 (45.30)* |
| BMI Analysis | 1,156 (97.72) | 1,184 (98.42) |
| Epilepsy and Sleep Disturbances | 1,156 (97.72)** | 1,196 (99.42)** |
| Health Outcomes Analysis | 1,110 (93.83)*** | 1,144 (95.10)*** |

Note: All exclusions are due to missing data unless specified below

*Only those who reported a Dietary Restriction could be included in this analysis

**Individuals excluded with syndromes that may increase risk of health outcomes

***Individuals excluded due to syndromes that may increase risk of health outcomes or missing data

Regarding specific sleep disturbances, non-responders to this question were assumed to have no sleep disturbances. We conducted a sensitivity analysis where we excluded any individual who had missing data for any of the other questions inquiring about diet, sleep, or exercise (n = 187 excluded). We used z-tests to confirm that the results do not change. Details of this sensitivity analysis is provided in Supplementary Table 3.

Similarly, non-responders to the question about dietary restrictions were assumed to have no dietary restrictions in our main analysis. We performed a sensitivity analysis excluding all individuals who had missing data for the dietary restriction question (n = 171). Again, we used z-tests to confirm that the results did not change. Details of this sensitivity analysis is provided in Supplementary Table 3.

**Supplementary Results:**

As noted in the manuscript, our results suggested that reduced sleep duration and increased rates of sleep disturbances seen among autistic adults could not be fully accounted for by epilepsy or seizure disorders. The full results from this model are provided below in Supplementary Table 2.

**Supplementary Table 2: Lack of effect when controlling for Epilepsy/ Seizure Disorder on the risk of Sleep Disturbances and Sleep Duration among Autistic vs. Non-Autistic Adults**

| Conditions | ß1 | SE1 | ß2 | SE2 | Z-test p-value |
| --- | --- | --- | --- | --- | --- |
| Sleeping Six Hours Per Night | -0.484 | 0.118 | -0.495 | 0.119 | 0.945 |
| Difficulty Falling Asleep | 0.700 | 0.119 | 0.677 | 0.119 | 0.892 |
| Difficulty Staying Asleep | 0.799 | 0.112 | 0.818 | 0.113 | 0.903 |
| Sleepwalking | 0.456 | 0.152 | 0.410 | 0.153 | 0.830 |
| Sleep Talking | 0.213 | 0.112 | 0.211 | 0.112 | 0.989 |
| Bedwetting | 0.710 | 0.178 | 0.632 | 0.181 | 0.756 |
| Frequent Night Terrors | 1.294 | 0.151 | 1.270 | 0.152 | 0.912 |
| Excessive Drowsiness | 0.841 | 0.107 | 0.806 | 0.108 | 0.821 |
| Narcolepsy | 1.227 | 0.491 | 1.161 | 0.495 | 0.926 |
| Sleep Apnea | 0.692 | 0.232 | 0.695 | 0.233 | 0.995 |

ß1 and SE1 refer to the original model; ß2 and SE2 refer to the model which includes presence of epilepsy/ seizure disorder as a covariate; z-test p-value provides the likelihood that the two models are significantly different

Note: values shown are rounded

As noted above, Supplementary Table 3 provide information on sensitivity analyses conducted as part of the study, to address pauses in survey collection and missingness due to non-response. There were no significant differences in any of these sensitivity analyses and full results are provided below.

**Supplementary Table 3: Sensitivity Analysis for Time Point**

| Conditions | ß1 | SE1 | ß2 | SE2 | Z-test  p-value |
| --- | --- | --- | --- | --- | --- |
| Exercising At Least Once Per Week | -0.416 | 0.112 | -0.430 | 0.115 | 0.929 |
| Meeting Weekly Exercise Goals | -0.504 | 0.117 | -0.513 | 0.120 | 0.957 |
| Meeting Fruit and Vegetable Goals | -0.518 | 0.106 | -0.490 | 0.108 | 0.851 |
| Eating High Calorie Foods | 0.271 | 0.114 | 0.220 | 0.118 | 0.757 |
| Meeting Daily Water Goals | 0.189 | 0.122 | 0.208 | 0.125 | 0.915 |
| Drinking High Sugar Beverages | 0.374 | 0.205 | 0.340 | 0.210 | 0.906 |
| Drinking Caffeinated Beverages | -0.309 | 0.111 | -0.277 | 0.114 | 0.841 |
| Vegan/ Vegetarian | 0.446 | 0.130 | 0.426 | 0.134 | 0.916 |
| Lactose Free | 0.937 | 0.176 | 0.958 | 0.181 | 0.932 |
| Nut Free | 0.693 | 0.337 | 0.662 | 0.345 | 0.947 |
| Gluten Free | 0.892 | 0.185 | 0.975 | 0.189 | 0.752 |
| Soy Free | 0.684 | 0.355 | 0.627 | 0.364 | 0.910 |
| No Fish | 0.755 | 0.264 | 0.704 | 0.270 | 0.893 |
| Other Dietary Restriction | 0.502 | 0.131 | 0.487 | 0.135 | 0.936 |
| No Dietary Restriction | -0.457 | 0.104 | -0.470 | 0.107 | 0.930 |
| Dietary Restriction Due to Allergy | 0.738 | 0.158 | 0.722 | 0.162 | 0.944 |
| Sleeping At least Six Hours Per Night | -0.460 | 0.118 | -0.481 | 0.122 | 0.905 |
| Difficulty Falling Asleep | 0.691 | 0.118 | 0.677 | 0.121 | 0.933 |
| Difficulty Staying Asleep | 0.803 | 0.112 | 0.758 | 0.114 | 0.780 |
| Sleepwalking | 0.451 | 0.151 | 0.500 | 0.155 | 0.820 |
| Sleep Talking | 0.201 | 0.111 | 0.198 | 0.114 | 0.988 |
| Bedwetting | 0.712 | 0.178 | 0.695 | 0.182 | 0.947 |
| Frequent Night Terrors | 1.298 | 0.151 | 1.309 | 0.154 | 0.959 |
| Excessive Drowsiness | 0.834 | 0.107 | 0.812 | 0.110 | 0.888 |
| Narcolepsy | 1.208 | 0.489 | 1.221 | 0.496 | 0.985 |
| Sleep Apnea | 0.647 | 0.230 | 0.707 | 0.235 | 0.855 |
| Underweight (Model 2) | 0.836 | 0.222 | 0.796 | 0.228 | 0.900 |
| Normal Range (Model 2) | -0.440 | 0.088 | -0.419 | 0.091 | 0.867 |
| Overweight (Model 2) | 0.091 | 0.099 | 0.073 | 0.104 | 0.902 |
| Obese (Model 2) | 0.319 | 0.102 | 0.325 | 0.107 | 0.969 |
| Underweight (Model 3) | 0.740 | 0.236 | 0.689 | 0.242 | 0.879 |
| Normal Range (Model 3) | -0.364 | 0.094 | -0.352 | 0.097 | 0.924 |
| Overweight (Model 3) | 0.115 | 0.105 | 0.102 | 0.109 | 0.935 |
| Obese (Model 3) | 0.202 | 0.112 | 0.218 | 0.116 | 0.919 |
| Female Cardiovascular Conditions (Model 2) | 0.366 | 0.152 | 0.371 | 0.159 | 0.982 |
| Male Cardiovascular Conditions (Model 2) | 0.422 | 0.203 | 0.503 | 0.214 | 0.784 |
| Female Diabetic Conditions (Model 2) | 0.667 | 0.222 | 0.799 | 0.229 | 0.677 |
| Male Diabetic Conditions (Model 2) | 0.508 | 0.340 | 0.576 | 0.363 | 0.891 |
| Female Cardiovascular Conditions (Model 3) | 0.368 | 0.154 | 0.376 | 0.161 | 0.971 |
| Male Cardiovascular Conditions (Model 3) | 0.409 | 0.203 | 0.486 | 0.215 | 0.795 |
| Female Diabetic Conditions (Model 3) | 0.626 | 0.223 | 0.765 | 0.232 | 0.666 |
| Male Diabetic Conditions (Model 3) | 0.518 | 0.341 | 0.585 | 0.363 | 0.892 |
| Female Cardiovascular Conditions (Model 4) | 0.364 | 0.167 | 0.364 | 0.173 | 0.999 |
| Male Cardiovascular Conditions (Model 4) | 0.346 | 0.223 | 0.409 | 0.234 | 0.846 |
| Female Diabetic Conditions (Model 4) | 0.839 | 0.250 | 0.975 | 0.257 | 0.706 |
| Male Diabetic Conditions (Model 4) | 0.430 | 0.384 | 0.446 | 0.413 | 0.977 |

ß1 and SE1 refer to the original model; ß2 and SE2 refer to the model which includes Time period as a covariate; z-test p-value provides the likelihood that the two models are significantly different

Note: values shown are rounded

**Survey Design:**

We have provided images of the questionnaire to display how the relevant questions regarding diet, exercise, sleep, BMI and physical health outcomes appeared to participants.

First, we asked participants to self-report their height and weight. We then converted all reported participant weights into kilograms and all reported participant heights into meters. Finally, we used the formula kg/m^2^ to derive individuals’ BMI (as was specified by the WHO in publicly available documentation, <https://www.who.int/dietphysicalactivity/childhood_what/en/>). In order to be inclusive towards individuals from all nationalities (and measurement systems), we allowed participants to report any measurement of height and weight with the units specified:

**Figure 1: Participants’ Height and Weight**

<INSERT SUPPLEMENTARY MATERIAL, FIGURE 1 HERE>

Below we have included an image of the survey questions pertaining to exercise patterns, including measures of exercise frequency and duration. From these, we derived the binary and categorical variables related to exercise goals. In order to provide the most conservative analysis, we always assumed that participants engaged in the maximum exercise specified within the categories when determining if they met exercise goals. For example, if an individual reported that they engage in exercise ‘two to three times per week’ and participated on average for ’15-30minutes’, we would have calculated that this individual participated in 90 minutes of exercise per week on average (which is greater than 75 minutes per week, which was our minimum exercise ‘goal’).

**Figure 2: Exercise Frequency and Duration Questions**

<INSERT SUPPLEMENTARY MATERIAL, FIGURE 2 HERE>

Below we have included an image of the survey questions pertaining to sleep duration and sleep disturbances. As noted in the main text of the article, we did not include a ‘None of the Above’ option for the question pertaining to sleep disturbances. For this reason, we conducted a sensitivity analysis and further information about this is available in Supplementary Table 3.

**Figure 3: Sleep Duration and Sleep Disturbances Questions**

<INSERT SUPPLEMENTARY MATERIAL, FIGURE 3 HERE>

Below we have included images of the survey questions pertaining to frequency of meeting fruit and vegetable goals (at least 5 servings of fruit or vegetables per day) as measured in days per week, frequency of eating high calorie foods as measured in days per week, whether the participant has any dietary restrictions, and whether any dietary restriction is due to an allergy. As a note, the allergy question only appeared if participants did not select the ‘No Dietary Restriction’ option provided in the previous question. Although there was a ‘No Dietary Restriction’ option provided in this question, we assumed that individuals who did not respond to the question also did not have a dietary restriction and simply did not see this option. Thus, as noted in the main text of the article, we conducted a sensitivity analysis on this question and further information about this is available in Supplementary Table 3.

**Figure 4: Fruit and Vegetable Goals, High Calorie Foods, and Dietary Restriction Questions**

<INSERT SUPPLEMENTARY MATERIAL, FIGURE 4 HERE>

**Figure 5: Dietary Restriction Due to an Allergy Question**

<INSERT SUPPLEMENTARY MATERIAL, FIGURE 5 HERE>

Below we have included an image of the survey questions related to beverages, specifically related to water, high sugar beverages, and caffeinated beverages:

**Figure 6: Water, High Sugar Beverages, and Caffeinated Beverages Questions**

<INSERT SUPPLEMENTARY MATERIAL, FIGURE 6 HERE>

Regarding questions related to physical health outcomes, participants were first directed to the question shown in Figure 7 below, and were asked to identify the relevant categories for their personal medical history:

**Figure 7: List of Condition Categories**

<INSERT SUPPLEMENTARY MATERIAL, FIGURE 7 HERE>

Follow-up questions with list of conditions would only appear for participants who selected a particular category. Participants were able to select two broad categories that related to cardiovascular conditions, Heart Condition or Risk of Stroke. Both of these conditions led to the same list of conditions related to cardiovascular health; this follow-up question is shown below in Figure 8:

**Figure 8: List of Cardiovascular Conditions**

<INSERT SUPPLEMENTARY MATERIAL, FIGURE 8 HERE>

Participants that selected the category for Prediabetes or Diabetes were directed to the following list of conditions, shown in Figure 9:

**Figure 9: List of Diabetic Conditions**

<INSERT SUPPLEMENTARY MATERIAL, FIGURE 9 HERE>

We corrected coding as necessary, to ensure that any condition listed throughout the survey (including information listed in any questions relating to syndromes, disabilities, autoimmune conditions, and free-text questions) was coded in the appropriate category. In addition, we corrected coding in order to meet our criteria for non-communicable diseases; for example, individuals who selected the broad category of ‘Heart Condition’ but only listed a particular infection and no other cardiovascular conditions were re-coded to note that they did not possess a cardiovascular condition. While the vast majority of respondents coded all conditions correctly, we wanted to ensure that conditions listed anywhere by participants were associated with the correct category.

Below we have included images of the questions related to Family History regarding cardiovascular and diabetic conditions. For ethical reasons, we did not ask participants to disclose any demographic information about their relatives; however, as individuals were asked to only report on first-degree biological relatives, we would assume that some features such as ethnicity would be similar to the participant completing the survey.

**Figure 10: Cardiovascular Family History Question**

<INSERT SUPPLEMENTARY MATERIAL, FIGURE 10 HERE>

**Figure 11: Diabetic Family History Question**

<INSERT SUPPLEMENTARY MATERIAL, FIGURE 11 HERE>
